# Reinvestigation of KMg_1/3_Nb_2/3_OPO_4_ 1

**DOI:** 10.1107/S1600536810004617

**Published:** 2010-02-10

**Authors:** Artem A. Babaryk, Vladimir Bon, Igor V. Zatovsky, Nikolay S. Slobodyanik, Vasily Pekhnyo

**Affiliations:** a Inorganic Chemistry Department, Faculty of Chemistry, Taras Shevchenko Kyiv National University, Kyiv, Ukraine; b Institute of General and Inorganic Chemistry, NAS Ukraine, prosp. Palladina 32/34, 03680 Kyiv, Ukraine

## Abstract

The crystal structure of potassium magnesium niobium oxide phosphate, KMg_1/3_Nb_2/3_OPO_4_, which was described in the space group *P*4_3_22 [McCarron & Calabrese, (1993 ▶). *J. Solid State Chem.* **102**, 354–361], has been redetermined in the revised space group *P*4_1_. Accordingly, the assignment of the space group *P*4_3_22 and, therefore, localization of K at a single half-occupied position, as noted in the previous study, proved to be an artifact. As a consequence, two major and two minor positions of K are observed due to the splitting along [001], as first noted for KTiOPO_4_ structure analogues. It has been shown that the geometry of the {*M* ^II^_1/3_Nb_2/3_O_6/2_}_∞_ framework is almost unaffected by the lowering of symmetry.

## Related literature

For the previous study of the title compound, see: McCarron & Calabrese (1993 ▶). For K-splitting in KTiOPO_4_ (KTP) isostructures, see: Belokoneva *et al.* (1997 ▶); Streltsov *et al.* (1998 ▶); Delarue *et al.* (1999 ▶); Nordborg (2000 ▶); Norberg & Ishizawa (2005 ▶) and in Nb-substituted KTP, see: Alekseeva *et al.* (2003 ▶); Dudka *et al.* (2005 ▶). For tetra­gonal aliovalent analogues related to KTiOPO_4_, see: Peuchert *et al.* (1995 ▶); Babaryk *et al.* (2007*b* ▶). For the relationship between the crystal symmetry class and non-zero χ^(2)^ coefficients, see: Authier (2003 ▶); Babaryk *et al.* (2007*a* ▶). For method used to determine the absolute structure, see: Flack & Bernardinelli (2000 ▶). 

## Experimental

### 

#### Crystal data

K_0.96_(Mg_0.32_Nb_0.68_O)(PO_4_)
*M* *_r_* = 219.46
Tetragonal, 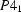

*a* = 6.5261 (1) Å
*c* = 10.8427 (4) Å
*V* = 461.79 (2) Å^3^
*Z* = 4
Mo *K*α radiation
μ = 3.02 mm^−1^
*T* = 293 K
0.3 × 0.3 × 0.3 mm

#### Data collection

Bruker APEXII diffractometer
Absorption correction: multi-scan [*SORTAV* (Blessing, 1995 ▶) and *SADABS* (Bruker, 2007 ▶)] *T*_min_ = 0.603, *T*_max_ = 0.746
2467 measured reflections
1036 independent reflections
1020 reflections with *I* > 2σ(*I*)
*R*_int_ = 0.017

#### Refinement

*R*[*F* ^2^ > 2σ(*F* ^2^)] = 0.025
*wR*(*F* ^2^) = 0.055
*S* = 1.20
1036 reflections
107 parameters
3 restraints
Δρ_max_ = 0.62 e Å^−3^
Δρ_min_ = −0.54 e Å^−3^
Absolute structure: Flack (1983 ▶), 336 Friedel pairs
Flack parameter: 0.08 (10)

### 

Data collection: *APEX2* (Bruker, 2007 ▶); cell refinement: *SAINT* (Bruker, 2007 ▶); data reduction: *SAINT*, and Blessing (1987 ▶, 1989 ▶); program(s) used to solve structure: *SHELXS97* (Sheldrick, 2008 ▶); program(s) used to refine structure: *SHELXL97* (Sheldrick, 2008 ▶); molecular graphics: *XP* in *SHELXTL* (Sheldrick, 2008 ▶) and *SLANT PLANE* in *WinGX* (Farrugia, 1999 ▶); software used to prepare material for publication: *SHELXTL*.

## Supplementary Material

Crystal structure: contains datablocks global, I. DOI: 10.1107/S1600536810004617/br2136sup1.cif

Structure factors: contains datablocks I. DOI: 10.1107/S1600536810004617/br2136Isup2.hkl

Additional supplementary materials:  crystallographic information; 3D view; checkCIF report

## Figures and Tables

**Table 1:** Comparison of bond lengths and angles (Å, °)

	This work	McCarron & Calabrese (1993 ▶)
P1—O1	1.531 (5)	1.533 (3)
P1—O2^i^	1.541 (5)	1.540 (3)
Mg(Nb)1—O1^iii^	2.116 (5)	2.124 (3)
Mg(Nb)1—O3	2.059 (3)	2.069 (3)
Mg(Nb)1—O5^iii^	1.888 (4)	1.890 (1)
O1—P1—O3	110.9 (2)	111.5 (1)
O3—P1—O2^i^	108.0 (2)	107.9 (2)
O5—Mg(Nb)1—O1^iii^	173.55 (14)	173.4 (1)
O5—Mg(Nb)1—O2^iii^	173.74 (14)	173.4 (1)
O4—Mg(Nb)1—O3	163.53 (10)	163.1 (2)

Symmetry codes: (i) 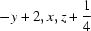; (iii) 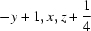.

**Fig. 1.**
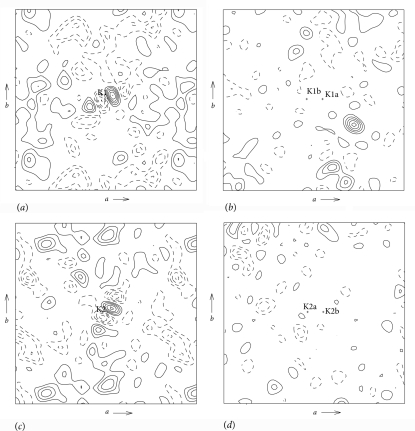
Comparison of difference Fourier maps through the K1 and K2 sites accounting (a, c) and without (b, d) the K-splitting. Contour intervals are 0.015 e/A3. The contours are drawn solid (positive) and dashed (negative) lines, respectively. Map scope is 4×4 Å.

**Fig. 2.**
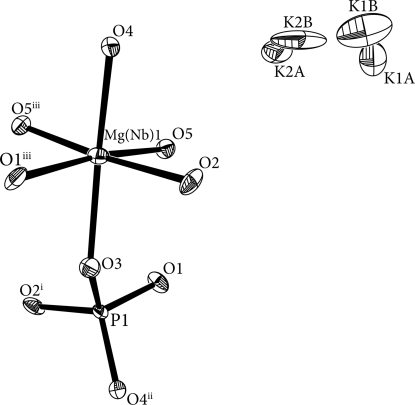
View of the the asymmetric unit. Displacement ellipsoids are drawn at the 50% probability level. [Symmetry codes: (i) -y+2, x, z+1/4 (ii) x+1, y, z; (iii) -y+1, x, z+1/4].

**Fig. 3.**
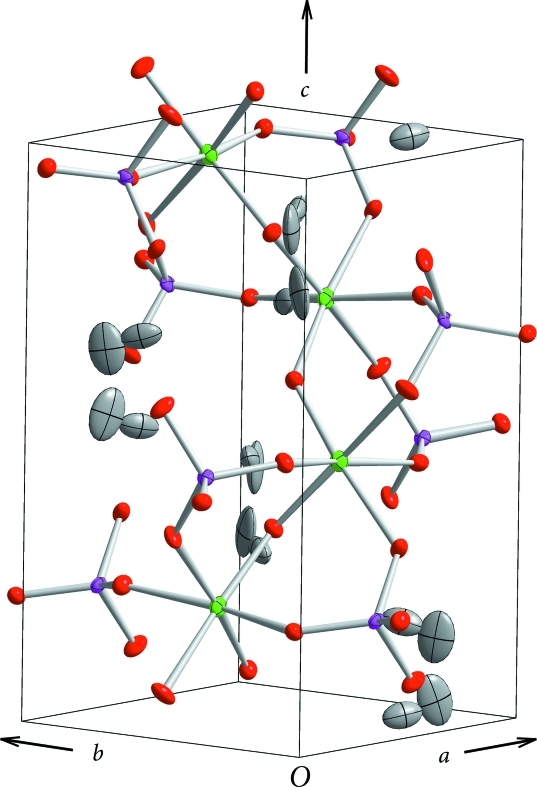
View of the the cell unit. Colour codes: oxygen is red, magnesium and niobium are green, phosphorus is purple, potassium is grey.

**Fig. 4.**
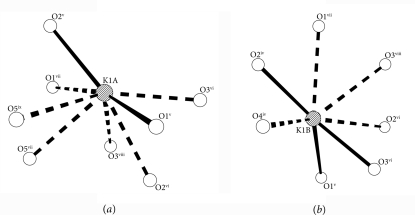
Oxygen cages of K1A (a) and K1B (b). Short and long K–O contact are showed as solid lines and dashed lines, respecively. [Symmetry codes: (iv) y-1, -x+1, z-1/4; (v) y, -x+2, z-1/4;(vi) -x+1, -y+2, z-1/2; (vii) -x+1, -y+1, z-1/2; (viii) -y+1, x, z-3/4; (ix) y, -x+1, z-1/4

## supplementary crystallographic information

### Comment

Originally KMg_1/3_Nb_2/3_PO_5_ was described by McCarron III & Calabrese (1993) who determined that it crystallized in non-polar space group *P*4_3_22 (95) chosen on the assumption of the almost zero-order experimental value of second harmonic response (SHG) relatively to α-SiO_2_ as standard. The asymmetric part of the unit cell consists of one Mg(Nb)(.2.), P1 (.2.) and O1 (..2) possesing the two-fold axes while K1 occupies a general position with *q*=0.5. O2, O3 are full-occupied. K1 is distant from its equivalent position (*y*, *x*, 1/4-*z*) at 1.582 (7) Å. Also the authors reported about the absolute configuration assignment based on exceptionally on Δ*R*_w_(F)=0.15 as criterion. The title compound can be described as {Mg_1/3_Nb_2/3_O_6/2_}_∞_ mutually perpendicular octahedral chains running along the principal crystal axes. PO_4/2_ tetrahedra link {Mg_1/3_Nb_2/3_O_6/2_}_2_ fragments of adjacent chains via the common O vertices. K atoms reside in the tunnel cavities of the anionic framework parallel to the [001] direction. However, indication of non-zero SHG value contradicts the assighment of 422 space groups, which have nil components of the χ^(2)^-tensor (see Authier *et al.*, 2003). An attempt to revise this model was done earlier (Babaryk *et al.*, 2007a) for Mn- and Co-containing isostructural compounds leading to lower symmetry lattices [space group *P*4_1_ (76) or *P*4_3_ (78), depending on the Flack parameter] where K1 (4a) and K2 (4a) positions were treated as independent.

This work aims to unify the description of all isostructural series of K*M*^II^_1/3_Nb_2/3_OPO_4_ (*M* = Mg, Mn, Co) based on a K-split structural model. Improvements brought by this model were tested on Mg-containing isostructure of the series.

Unlike the previous work, all refiments were carried out on *F*^2^_obs_, the absolute structure of the title compound was estimated on the basis of the Flack parameter with the TWIN/BASF instruction (Sheldrick, 2008) by sparse-matrix least squares (Flack & Bernardinelli, 2000). The initial coordinates were taken from the Babaryk *et al.*, (2007a). The initial ratio of Mg and Nb occupancies was fixed at the ideal value 1/2 while occupancies of K(1) and K(2) were free and refined to the following convergence parameters: R = 0.038, wR = 0.082, S = 1.113 and residual electron densities Δρ_max_/Δρ_min_ = 1.41/-0.86 e Å^3^ [σ(Δρ) = 0.14 e/Å^3^]. The highest observed peak (1.41 e Å^3^) is distant from 0.92Å from K1 and the lowest one at a distance of 0.27 Å from K(2) (Fig. 1a,c) and corresponds to splitting along the c-axis that was earlier shown (Norberg *et al.*, 2005) for KTiOPO_4_ only in a case of synchrotron radiation experiments. In the modified model occupancies of K atoms were set of 0.25 equally and further refinement was performed with linear restraints on the occupancies to fit the charge balance. The refinement converged to R = 0.026, wR = 0.055, S = 1.195 and residual electron densities Δρ_max_/Δρ_min_ = 0.62/-0.54 e Å^3^ [σ(Δρ) = 0.11 e Å^3^] (Fig. 1 b,d). Comparison of result obtained on the previously reported model tested on the same dataset lead to less satisfactoral results R = 0.043, wR = 0.095, S =1.112 and Δρ_max_/Δρ_min_ = 1.23/-0.88 e Å^3^ [σ(Δρ) = 0.19 e Å^3^] in *P*4_1_22 (93) [Flack parameter *x* = 0.8 (10)]. The highest peak (1.23 e Å^3^) observed on the (*F*_o_-*F*_c_)-map distant from K1 at 0.95 Å.

The asymmetric and cell units are shown on the Fig. 2 and 3.

The comparison of P—O and Mg/Nb—O bond length and angles (Table 1) produce rather comparable values in this study and previous ones, thus, the geometry of {*M*^II^_1/3_Nb_2/3_O_6/2_}_∞_ is almost not affected by the lowering of symmetry. The major differences between the previously reported and this investigation are concerned with K-splittig phenomena. To the the best of our knowledge this is the first example among tetragonal KTP-analogues (Peuchert *et al.*, 1995; Babaryk *et al.*, 2007b) where it is observed. K-splitting phenomena for KTiOPO_4_ family of compounds is intesively studied over the last decade. Examples of splitting at room temperature for KTP-isostructures become more evident, verbi causa, RbSbOGeO_4_ (Belokoneva *et al.*, 1997), RbTiOAsO_4_ (Streltsov *et al.*, 2000), CsTiOAsO_4_ (Nordborg, 2000), KTiOPO_4_ (Norberg & Ishizawa, 2005) using synchrotron irradiation experiments. In case X-ray study of KTP such splitting was found only upon heating at 673 and 973 K (Delarue *et al.*, 1999). Insert of 7-11% at. of Nb into KTP allow to introduce the cation-split model for refinement and, noteworthy, occupation of minor K-position growth with increasing Nb-content in KTiOPO_4_ (Alekseeva *et al.*, 2003). Recent precision investigation (30 K) of 7% at. Nb-doped KTP crystals (Dudka *et al.*, 2005) showed K distribution over the split position is almost indentical to results obtained at room temperature. Thus, it is likely to introduce the K-split model for refinement of Nb-containing KTP analogues as well as it was applied above.

The final distribution of K over the positions K1A/K1B is equiproportional to K1B/K2B ones that are of ca. 7/3. The arrangement of K atoms is almost symmetrical, the distance between major K1A and minor K1B positions is 0.70 (2) Å that is almost equal to 0.69 (3) Å K(2 A)—K(2B) separation. The distance between major K1A and K2A positions is something larger [1.729 (8) Å] in counterpart of 1.21 (3) Å between K1B and K2B, while corresponding cross-distances are equal to 1.58 (3) Å. The discrepancies between the coordination of major positions K1A, K2A and minor K1B and K2B is also observed in this study similar to that reported earlier (Norberg & Ishizawa, 2005). Both K1A and K2A atoms formed eight K—O contacts (Fig. 4a) according to a scheme [2+6]: two of them are in a range of 2.592 (7)-2.713 (7) Å corresponding to ionic-covalent type and six are longer ones within the limits 2.862 (1)-3.138 (1) Å demonstrating ionic bonding type. In counterpart to this each K1B and K2B are closed with seven O atoms (Fig. 4b), three of them are being short [2.572 (1)-2.784 (2) Å], other three ones are longer [2.96 (3)-3.26 (4) Å]. The seventh contact is distinguished by the value of 3.37 (3) Å so it is hardly side with others.

One can conclude that the cation subblattice arrangement is silmilar either for KTP isostructures or its aliovalent analogues, however a measure of K-splitting "strength" depends on the charge of the polyvalent metal constituent.

### Experimental

For preparation of the title compound 1.072 (1) g of KPO_3_, 0.122 (1) g of MgO, 0.806 (1) g of Nb_2_O_5_ and 10.000 (1) g of K_2_Mo_2_O_7_ were mixed together and well grounded in an agate mortar. All the reagents were of analytical grade purity. The mixture was melted in a 50 ml platinum crucible at 1273 K. Melted solution was cooled at a rate 20 K×h^-1^ up to 1133 (5) K. Crystalline precipitate was freed from the liquid flux by decantation. The crystals were washed from the rest of the solidified component with hot 5%—aqueous (NaPO_3_)*_x_*. Crystalline phase consists of well-shaped colourless biaxial crystals (with size distribution from 0.2 to 0.4 mm of length) in major unless the rear pale-red prismatic crystals of K_5+x_Nb_8-x_Mg_x_P_5_O_34_ (unpublished results). It is likely to obtaine pure compound at more slow cooling of the initial melt.

Evaluation of the elements quantities was performed on an X-ray fluorescence energy dispersive spectrometer Elvax Light and established Mo presence at a level of 0.01% at., which has not been taken into account at the structure refinements. Exact compositions of title compound was found using a Spectroflame Modula ICP instrument. Analysis found: K, 12.12; Mg, 4.11; Nb, 8.57; P, 12.60; O, 63.94; Mo, 0.06%. K_0.96_Mg_0.32_Nb_0.68_PO_5_ requires: K, 12.06; Mg, 4.02; Nb, 8.54; P, 12.56; O, 62.81%.

### Crystal data

**Table:**

K_0.96_(Mg_0.32_Nb_0.68_O)(PO_4_)	*D*_x_ = 3.158 Mg m^−^^3^
*M**_r_* = 219.46	Mo *K*α radiation, λ = 0.71073 Å
Tetragonal, *P*4_1_	Cell parameters from 2061 reflections
Hall symbol: P 4w	θ = 3.1–30.6°
*a* = 6.5261 (1) Å	µ = 3.02 mm^−^^1^
*c* = 10.8427 (4) Å	*T* = 293 K
*V* = 461.79 (2) Å^3^	Block, colourless
*Z* = 4	0.3 × 0.3 × 0.3 mm
*F*(000) = 420.4	

### Data collection

**Table:**

Bruker APEXII diffractometer	1036 independent reflections
Radiation source: sealed x-ray tube	1020 reflections with *I* > 2σ(*I*)
graphite	*R*_int_ = 0.017
φ or ω oscillation scans	θ_max_ = 30.0°, θ_min_ = 3.1°
Absorption correction: multi-scan [*SORTAV* (Blessing, 1995) and *SADABS* (Bruker, 2007)]	*h* = −8→5
*T*_min_ = 0.603, *T*_max_ = 0.746	*k* = −9→5
2467 measured reflections	*l* = −7→15

### Refinement

**Table:**

Refinement on *F*^2^	Secondary atom site location: difference Fourier map
Least-squares matrix: full	*w* = 1/[σ^2^(*F*_o_^2^) + 1.2901*P*] where *P* = (*F*_o_^2^ + 2*F*_c_^2^)/3
*R*[*F*^2^ > 2σ(*F*^2^)] = 0.025	(Δ/σ)_max_ = 0.001
*wR*(*F*^2^) = 0.055	Δρ_max_ = 0.62 e Å^−^^3^
*S* = 1.20	Δρ_min_ = −0.54 e Å^−^^3^
1036 reflections	Extinction correction: *SHELXL*
107 parameters	Extinction coefficient: 0.0115 (15)
3 restraints	Absolute structure: Flack (1983), 336 Friedel pairs
Primary atom site location: isomorphous structure methods	Flack parameter: 0.08 (10)

### Special details

**Table:**

Geometry. All esds (except the esd in the dihedral angle between two l.s. planes) are estimated using the full covariance matrix. The cell esds are taken into account individually in the estimation of esds in distances, angles and torsion angles; correlations between esds in cell parameters are only used when they are defined by crystal symmetry. An approximate (isotropic) treatment of cell esds is used for estimating esds involving l.s. planes.
Refinement. Refinement of *F*^2^ against ALL reflections. The weighted *R*-factor wR and goodness of fit *S* are based on *F*^2^, conventional *R*-factors *R* are based on *F*, with *F* set to zero for negative *F*^2^. The threshold expression of *F*^2^ > σ(*F*^2^) is used only for calculating *R*-factors(gt) etc. and is not relevant to the choice of reflections for refinement. *R*-factors based on *F*^2^ are statistically about twice as large as those based on *F*, and *R*- factors based on ALL data will be even larger.

### Fractional atomic coordinates and isotropic or equivalent isotropic displacement parameters (Å^2^)

**Table:**

	*x*	*y*	*z*	*U*_iso_*/*U*_eq_	Occ. (<1)
O1	0.9717 (6)	0.5139 (5)	0.8485 (5)	0.0163 (9)	
O2	0.5141 (5)	0.9718 (6)	0.8260 (5)	0.0169 (10)	
O3	0.8102 (4)	0.7859 (4)	0.9827 (3)	0.0144 (7)	
O4	0.1898 (4)	0.7865 (4)	0.9406 (3)	0.0133 (7)	
O5	0.5433 (5)	0.5429 (5)	0.8361 (4)	0.0132 (6)	
P1	1.00000 (13)	0.65145 (13)	0.96154 (16)	0.01016 (19)	
K1A	0.2428 (18)	0.7800 (11)	0.5076 (6)	0.034 (3)	0.344 (17)
K1B	0.155 (4)	0.827 (2)	0.531 (3)	0.056 (7)	0.148 (16)
K2A	0.2198 (14)	0.758 (2)	0.6659 (7)	0.034 (3)	0.332 (19)
K2B	0.173 (3)	0.844 (5)	0.642 (3)	0.054 (7)	0.140 (18)
Mg1	0.49981 (6)	0.74099 (6)	0.96157 (9)	0.01355 (13)	0.3214 (12)
Nb1	0.49981 (6)	0.74099 (6)	0.96157 (9)	0.01355 (13)	0.6786 (12)

### Atomic displacement parameters (Å^2^)

**Table:**

	*U*^11^	*U*^22^	*U*^33^	*U*^12^	*U*^13^	*U*^23^
O1	0.0156 (15)	0.0163 (18)	0.017 (2)	0.0044 (12)	−0.0035 (17)	−0.0086 (13)
O2	0.0172 (19)	0.0154 (15)	0.018 (2)	0.0050 (12)	0.0065 (14)	0.0085 (17)
O3	0.0125 (12)	0.0158 (12)	0.015 (2)	0.0014 (10)	0.0002 (14)	−0.0028 (13)
O4	0.0126 (12)	0.0154 (12)	0.012 (2)	−0.0010 (10)	0.0003 (13)	0.0015 (13)
O5	0.0132 (14)	0.0124 (14)	0.0139 (13)	−0.0007 (8)	0.0017 (14)	−0.0026 (14)
P1	0.0071 (3)	0.0133 (4)	0.0101 (4)	0.0002 (3)	−0.0020 (4)	−0.0003 (5)
K1A	0.049 (5)	0.027 (2)	0.026 (3)	−0.022 (3)	−0.007 (2)	0.0018 (18)
K1B	0.044 (9)	0.019 (5)	0.10 (2)	−0.019 (5)	0.019 (10)	−0.006 (6)
K2A	0.026 (3)	0.049 (5)	0.027 (3)	−0.021 (3)	0.0017 (19)	−0.010 (2)
K2B	0.018 (6)	0.042 (10)	0.10 (2)	−0.016 (6)	−0.003 (7)	0.008 (11)
Mg1	0.00835 (18)	0.0162 (2)	0.0161 (2)	0.00006 (16)	−0.0009 (2)	0.0003 (3)
Nb1	0.00835 (18)	0.0162 (2)	0.0161 (2)	0.00006 (16)	−0.0009 (2)	0.0003 (3)

### Geometric parameters (Å, °)

**Table:**

P1—O1	1.531 (5)	K1A—O3^ix^	2.993 (7)
P1—O2^i^	1.541 (5)	K1A—O5^x^	3.002 (12)
P1—O3	1.535 (3)	K1A—O2^vii^	3.004 (9)
P1—O4^ii^	1.537 (3)	K1A—O5^viii^	3.138 (13)
Nb1—O1^iii^	2.116 (5)	K2A—O2^iv^	2.592 (7)
Nb1—O2	2.107 (5)	K2A—O1^vi^	2.709 (8)
Nb1—O3	2.059 (3)	K2A—O4^iv^	2.862 (10)
Nb1—O4	2.057 (3)	K2A—O2	2.941 (7)
Nb1—O5^iii^	1.888 (4)	K2A—O4	2.990 (7)
Nb1—O5	1.898 (4)	K2A—O5^x^	3.008 (14)
K1A—K1B	0.70 (2)	K2A—O1^xi^	3.012 (9)
K1A—K2B	1.58 (3)	K2A—O5	3.134 (15)
K1A—K2A	1.729 (8)	K1B—O2^iv^	2.572 (11)
K1B—K2B	1.21 (3)	K1B—O3^vii^	2.591 (13)
K2A—K2B	0.69 (3)	K1B—O1^vi^	2.784 (18)
K1B—K2A	1.58 (3)	K1B—O4^iv^	2.96 (3)
K1B—K2B^iv^	2.53 (2)	K1B—O1^viii^	3.09 (3)
K1B—K2A^iv^	2.89 (3)	K1B—O3^ix^	3.26 (3)
K1A—K2B^iv^	2.91 (3)	K1B—O2^vii^	3.37 (3)
K2A—K1B^v^	2.89 (3)	K2B—O1^vi^	2.574 (13)
K2B—K1B^v^	2.53 (2)	K2B—O1^xi^	3.37 (3)
K2B—K1A^v^	2.91 (3)	K2B—O2^iv^	2.773 (19)
K1A—O1^vi^	2.593 (6)	K2B—O2	3.10 (3)
K1A—O2^iv^	2.713 (7)	K2B—O3^vii^	2.97 (4)
K1A—O3^vii^	2.867 (9)	K2B—O4^iv^	2.586 (15)
K1A—O1^viii^	2.935 (7)	K2B—O4	3.26 (4)
			
O1—P1—O3	110.9 (2)	O2^iv^—K2A—O4	112.1 (3)
O1—P1—O4^ii^	108.4 (2)	O1^vi^—K2A—O4	105.9 (2)
O3—P1—O4^ii^	110.17 (14)	O4^iv^—K2A—O4	80.48 (18)
O1—P1—O2^i^	108.55 (17)	O2—K2A—O4	54.93 (16)
O3—P1—O2^i^	108.0 (2)	O2^iv^—K2A—O5^x^	83.3 (3)
O4^ii^—P1—O2^i^	110.8 (2)	O1^vi^—K2A—O5^x^	81.5 (2)
O1^vi^—K1A—O1^viii^	163.1 (5)	O4^iv^—K2A—O5^x^	142.9 (3)
O2^iv^—K1A—O1^viii^	52.22 (12)	O2—K2A—O5^x^	91.3 (3)
O3^vii^—K1A—O1^viii^	122.2 (3)	O4—K2A—O5^x^	111.9 (5)
O1^vi^—K1A—O3^ix^	112.3 (3)	O2^iv^—K2A—O1^xi^	63.26 (17)
O2^iv^—K1A—O3^ix^	106.1 (2)	O1^vi^—K2A—O1^xi^	154.5 (3)
O3^vii^—K1A—O3^ix^	80.42 (17)	O4^iv^—K2A—O1^xi^	58.02 (14)
O1^viii^—K1A—O3^ix^	55.08 (15)	O2—K2A—O1^xi^	102.4 (3)
O1^vi^—K1A—O5^x^	83.5 (3)	O4—K2A—O1^xi^	48.97 (16)
O2^iv^—K1A—O5^x^	81.4 (2)	O5^x^—K2A—O1^xi^	102.8 (5)
O3^vii^—K1A—O5^x^	143.1 (3)	O2^iv^—K2A—O5	109.7 (5)
O1^viii^—K1A—O5^x^	91.1 (3)	O1^vi^—K2A—O5	88.5 (3)
O3^ix^—K1A—O5^x^	111.7 (4)	O4^iv^—K2A—O5	131.6 (3)
O1^vi^—K1A—O2^vii^	63.37 (16)	O2—K2A—O5	54.9 (2)
O2^iv^—K1A—O2^vii^	154.8 (3)	O4—K2A—O5	59.1 (2)
O3^vii^—K1A—O2^vii^	57.92 (13)	O5^x^—K2A—O5	53.5 (3)
O1^viii^—K1A—O2^vii^	102.6 (3)	O1^xi^—K2A—O5	74.9 (3)
O3^ix^—K1A—O2^vii^	49.03 (15)	O1^vi^—K2B—O4^iv^	156.9 (14)
O5^x^—K1A—O2^vii^	102.7 (4)	O1^vi^—K2B—O2^iv^	139.1 (10)
O1^vi^—K1A—O5^viii^	110.0 (4)	O4^iv^—K2B—O2^iv^	61.3 (3)
O2^iv^—K1A—O5^viii^	88.7 (2)	O1^vi^—K2B—O3^vii^	58.7 (6)
O3^vii^—K1A—O5^viii^	131.4 (3)	O4^iv^—K2B—O3^vii^	102.9 (12)
O1^viii^—K1A—O5^viii^	54.89 (19)	O2^iv^—K2B—O3^vii^	123.5 (13)
O3^ix^—K1A—O5^viii^	58.8 (2)	O1^vi^—K2B—O2	51.1 (4)
O5^x^—K1A—O5^viii^	53.5 (2)	O4^iv^—K2B—O2	126.3 (11)
O2^vii^—K1A—O5^viii^	74.8 (3)	O2^iv^—K2B—O2	137.1 (15)
O2^iv^—K1B—O3^vii^	157.4 (12)	O3^vii^—K2B—O2	97.3 (5)
O2^iv^—K1B—O1^vi^	138.6 (9)	O1^vi^—K2B—O4	102.0 (9)
O3^vii^—K1B—O1^vi^	61.2 (3)	O4^iv^—K2B—O4	79.7 (8)
O2^iv^—K1B—O4^iv^	58.8 (5)	O2^iv^—K2B—O4	100.2 (10)
O3^vii^—K1B—O4^iv^	103.1 (10)	O3^vii^—K2B—O4	132.0 (8)
O1^vi^—K1B—O4^iv^	123.3 (11)	O2—K2B—O4	50.9 (6)
O2^iv^—K1B—O1^viii^	51.2 (4)	O5^iii^—Nb1—O5	93.97 (9)
O3^vii^—K1B—O1^viii^	126.4 (11)	O5^iii^—Nb1—O4	91.86 (13)
O1^vi^—K1B—O1^viii^	137.0 (13)	O5—Nb1—O4	99.56 (15)
O4^iv^—K1B—O1^viii^	97.5 (5)	O5^iii^—Nb1—O3	99.36 (15)
O2^iv^—K1B—O3^ix^	102.2 (8)	O5—Nb1—O3	91.70 (13)
O3^vii^—K1B—O3^ix^	79.6 (7)	O4—Nb1—O3	163.53 (10)
O1^vi^—K1B—O3^ix^	100.1 (8)	O5^iii^—Nb1—O2	173.74 (14)
O4^iv^—K1B—O3^ix^	132.4 (8)	O5—Nb1—O2	88.9 (2)
O1^viii^—K1B—O3^ix^	51.0 (5)	O4—Nb1—O2	82.15 (13)
O2^iv^—K2A—O1^vi^	141.9 (3)	O3—Nb1—O2	86.13 (14)
O2^iv^—K2A—O4^iv^	59.99 (17)	O5^iii^—Nb1—O1^iii^	88.8 (2)
O1^vi^—K2A—O4^iv^	130.3 (6)	O5—Nb1—O1^iii^	173.55 (14)
O2^iv^—K2A—O2	162.7 (6)	O4—Nb1—O1^iii^	86.18 (13)
O1^vi^—K2A—O2	52.19 (12)	O3—Nb1—O1^iii^	82.08 (13)
O4^iv^—K2A—O2	122.2 (3)	O2—Nb1—O1^iii^	89.02 (10)

Symmetry codes: (i) −*y*+2, *x*, *z*+1/4; (ii) *x*+1, *y*, *z*; (iii) −*y*+1, *x*, *z*+1/4; (iv) *y*−1, −*x*+1, *z*−1/4; (v) −*y*+1, *x*+1, *z*+1/4; (vi) *y*, −*x*+2, *z*−1/4; (vii) −*x*+1, −*y*+2, *z*−1/2; (viii) −*x*+1, −*y*+1, *z*−1/2; (ix) −*y*+1, *x*, *z*−3/4; (x) *y*, −*x*+1, *z*−1/4; (xi) *x*−1, *y*, *z*.

### Table 1 Comparison of bond lengths and angles (Å, °)

**Table:**

	This work	McCarron & Calabrese (1993)
P1—O1	1.531 (5)	1.533 (3)
P1—O2^i^	1.541 (5)	1.540 (3)
Mg(Nb)1—O1^iii^	2.116 (5)	2.124 (3)
Mg(Nb)1—O3	2.059 (3)	2.069 (3)
Mg(Nb)1—O5^iii^	1.888 (4)	1.890 (1)
O1—P1—O3	110.9 (2)	111.5 (1)
O3—P1—O2^i^	108.0 (2)	107.9 (2)
O5—Mg(Nb)1—O1^iii^	173.55 (14)	173.4 (1)
O5—Mg(Nb)1—O2^iii^	173.74 (14)	173.4 (1)
O4—Mg(Nb)1—O3	163.53 (10)	163.1 (2)

Symmetry codes: (i) -y+2, x, z+1/4; (iii) -y+1, x, z+1/4].
